# Impact of Histological Type and Grade on the Diagnostic Accuracy of Intraoperative Frozen Section for Detecting Breast Cancer Metastasis to Axillary Sentinel Lymph Nodes

**DOI:** 10.7759/cureus.16146

**Published:** 2021-07-03

**Authors:** Atif A Hashmi, Rubina Riaz, Shamail Zia, Hiba Shahid, Umair Arshad Malik, Rabeet Khan, Muhammad Irfan, Farozaan Shamail, Fazail Zia, Muhammad Ghani Asif

**Affiliations:** 1 Pathology, Liaquat National Hospital and Medical College, Karachi, PAK; 2 Pathology, Fazaia Medical College, Air University, Islamabad, PAK; 3 Pathology, Ziauddin University, Karachi, PAK; 4 Internal Medicine, Liaquat National Hospital and Medical College, Karachi, PAK; 5 Internal Medicine, Aga Khan University, Karachi, PAK; 6 Internal Medicine, Buckinghamshire Healthcare NHS Trust, Aylesbury, GBR; 7 Statistics, Liaquat National Hospital and Medical College, Karachi, PAK; 8 Pathology, Jinnah Sindh Medical University, Karachi, PAK; 9 Pathology, Multan Medical and Dental College, Multan, PAK

**Keywords:** intraoperative frozen section, sentinel lymph node (sln), breast carcinoma, infiltrating ductal carcinoma, infiltrating lobular carcinoma

## Abstract

Introduction

Intraoperative sentinel lymph node (SLN) evaluation is the standard of care in patients with clinically node-negative breast cancer. The most common histological subtype of breast carcinoma is invasive ductal carcinoma (IDC), followed by invasive lobular carcinoma (ILC). Alternatively, histological grades vary from grades G1 to G3. Therefore, in this study, we evaluated the diagnostic accuracy of frozen section (FS) for detecting breast cancer metastasis to SLNs with respect to histological subtypes and grades.

Methods

A retrospective observational study was conducted in the Department of Histopathology at Liaquat National Hospital and Medical College, Pakistan, from January 2013 till December 2020, over a duration of eight years. A total of 540 cases of primary breast cancer, undergoing upfront breast surgery were included in the study. Intraoperatively, SLNs were identified and sent for FS. After FS reporting, the remaining tissue was submitted for final (paraffin) section examination after formalin fixation, and results of FS and final (paraffin) sections were compared.

Results

The mean age of the patients included in the study was 52.05±12.42 years, and the median number of SLNs was three (ranging from one to 14). The overall sensitivity, specificity, positive predictive value, negative predictive value, and diagnostic accuracy of intraoperative FS were 88.2%, 100%, 100%, 92.5%, and 95.2%, respectively. The sensitivity of FS for IDC was 88.3%, whereas it was 85.7% for ILC. Alternatively, the sensitivity of FS for grade G1, G2, and G3 tumors was 78.3%, 91.5%, and 90.2%, respectively. The false-negative rate for grade G1 tumors was 21.7%, which was higher than G2 and G3 tumors (8.5% and 9.8%, respectively). Similarly, the false-negative rate for cases where the number of SLNs was more than three was only 5.4%, which was lower than cases with a single and two to three SLNs sent on FS (23.1 and 14.7%, respectively).

Conclusion

The sensitivity of intraoperative FS for detecting ILC metastasis to axillary SLNs was not substantially different from IDC; however, histological grade affects the sensitivity of FS diagnosis, with lower-grade tumors having low sensitivity. Moreover, increasing the number of SLNs sent intraoperatively on FS improves the sensitivity of FS for detecting breast cancer metastasis to axillary SLNs.

## Introduction

Breast cancer is one of the most common cancers among women in Southeast Asia, with an increasing number of cases at a young age [1]. Breast cancer is a heterogeneous disease with many histological and molecularly distinct subtypes. Apart from the common subtypes, many rare special subtypes of breast carcinoma were defined [2-3]. Alternatively, these different subtypes vary with respect to hormone receptor and human epidermal growth factor receptor 2 (HER2/neu) expressions [4-5]. Apart from cancer-related morbidity, chemotherapy for breast cancer is also associated with a significant number of complications [6-7]. Surgical management of breast cancer has evolved over a period of years, with respect to the axillary lymph node dissection. The most important prognostic factor in breast cancer is axillary lymph node metastasis. Alternatively, axillary lymph node dissection leads to increased morbidity in cancer patients. To overcome this problem, intraoperative sentinel lymph node (SLN) evaluation has become a standard of care in patients with clinically node-negative breast cancer [8]. Negative SLN on frozen section (FS) spares a patient from unnecessary axillary lymph node dissection.

Previous studies have confirmed a good diagnostic accuracy of FS for detecting axillary lymph node metastasis [9]. However, breast cancer is a heterogeneous disease with different histological subtypes and grades [10]. It is unclear if grade and histological type affect the diagnostic accuracy of FS for detecting metastatic carcinoma in SLNs. The most common histological subtype of breast carcinoma is invasive ductal carcinoma (IDC), followed by invasive lobular carcinoma (ILC). Alternatively, histological grades vary from grade G1 to G3. Therefore, in this study, we evaluated the diagnostic accuracy of FS for detecting breast cancer metastasis in SLNs with respect to histological subtypes and grades.

## Materials and methods

A retrospective observational study was conducted in the Department of Histopathology at Liaquat National Hospital and Medical College, Pakistan, from January 2013 till December 2020, over a duration of eight years. A total of 540 cases of primary breast cancer undergoing upfront breast surgery were included in the study. All these cases had clinically negative axillary lymph nodes and biopsy-proven breast carcinoma. Cases with a histological diagnosis of either IDC or ILC of any grade were included in the study. Cases with neoadjuvant chemotherapy or radiation were excluded from the study. Immunohistochemical (IHC) staining for E-cadherin was performed on trucut biopsy to confirm the diagnosis of ILC. Intraoperatively, SLNs were identified and sent for FS. The number and size of SLNs were recorded, and SLNs were sectioned at a 2 mm interval perpendicular to the long axis of the lymph nodes and submitted for FS. Three step levels were examined on FS after hematoxylin and eosin (H & E) staining. The remaining tissue was submitted for final (paraffin) section examination after formalin fixation. One H & E-stained section was examined on the final (paraffin) section and IHC staining was performed where necessary using cytokeratin (CKAE1/AE3) stain. The diagnosis of FS was compared with the final (paraffin) section for analysis.

Based on the diagnosis of FS, a decision regarding axillary dissection was made. For patients undergoing mastectomy, any positive SLN with macrometastasis (>2 mm metastatic deposit) was followed by axillary dissection. For cases undergoing breast conservation surgery, three or more positive SLNs with macrometastasis were followed by axillary dissection. In the remaining cases, axillary dissection was not performed.

After SLN resection, definitive breast surgery (lumpectomy or modified radical mastectomy) was performed, and specimens were sent to histopathology laboratory in formalin. After overnight formalin fixation, gross examination of the specimen was done and tumor size was measured. The representative sections were submitted from the tumor, resection margins, normal breast tissue, and axillary lymph nodes [11-14].

Data analysis was performed using the Statistical Package for the Social Sciences (Version 26.0, IBM Corp, Armonk, NY). Sensitivity, specificity, positive predictive value (PPV), negative predictive value (NPV), and diagnostic accuracy were calculated for FS diagnosis by 2 × 2 tables using the final (paraffin) section diagnosis as the gold standard.

## Results

The mean age of the patients included in the study was 52.05±12.42 years, and the median number of SLNs was three (ranging from one to 14). The most common tumor grade was G3 (41.9%). A total of 40.7% SLNs were positive for metastatic carcinoma on final (paraffin) histology (Table 1).

**Table 1:** Clinicopathological features of the population under study SD, standard deviation; IDC, infiltrating ductal carcinoma; ILC, infiltrating lobular carcinoma

Clinicopathological features	Values
Age (years), mean±SD	52.05±12.42
Age groups	
≤50 years, n (%)	288 (53.3)
>50 years, n (%)	252 (46.7)
Number of SLNs, median (range)	3 (1-14)
Histological type	
IDC, n (%)	468 (86.7)
ILC, n (%)	72 (13.3)
Grade	
Grade 1, n (%)	100 (18.5)
Grade 2, n (%)	214 (39.6)
Grade 3, n (%)	226 (41.9)
Tumor size	
≤2 cm, n (%)	68 (12.6)
2.1–5 cm, n (%)	416 (77)
> 5cm, n (%)	56 (10.4)
Type of surgery	
Lumpectomy, n (%)	220 (40.7)
Modified radical mastectomy, n (%)	198 (36.7)
Simple mastectomy, n (%)	122 (22.6)
Laterality	
Right, n (%)	332 (61.5)
Left, n (%)	208 (38.5)
Frozen section diagnosis	
Positive for metastatic carcinoma, n (%)	194 (35.6)
Negative for metastatic carcinoma, n (%)	346 (64.4)
Final (paraffin) section diagnosis	
Positive for metastatic carcinoma, n (%)	220 (40.7)
Negative for metastatic carcinoma, n (%)	320 (59.3)

The overall sensitivity, specificity, positive predictive value (PPV), negative predictive value (NPV), and diagnostic accuracy of intraoperative FS were 88.2%, 100%, 100%, 92.5%, and 95.2%, respectively (Table 2).

**Table 2:** Comparison of frozen section diagnosis with final (paraffin) section diagnosis for the detection of sentinel lymph node metastasis PPV, positive predictive value; NPV, negative predictive value

Frozen section diagnosis	Final (paraffin) section diagnosis			Sensitivity	Specificity	PPV	NPV	Diagnostic accuracy
	Positive	Negative	Total					
Positive	194	0	194	88.2%	100%	100%	92.5%	95.2%
Negative	26	320	346					
Total	220	320	540					

Table 3 compares the sensitivity, specificity, PPV, NPV, and diagnostic accuracy of intraoperative FS with respect to histological type. The sensitivity of FS for IDC was 88.3%, whereas it was 85.7% for ILC. Similarly, the diagnostic accuracy of intraoperative FS for IDC and ILC was 94.9% and 97.2%, respectively.

**Table 3:** Comparison of frozen section diagnosis with final (paraffin) section diagnosis for the detection of sentinel lymph node metastasis with respect to histological type IDC, infiltrating ductal carcinoma; ILC, infiltrating lobular carcinoma; PPV, positive predictive value; NPV, negative predictive value

Histological type	Frozen section diagnosis	Final (paraffin) section diagnosis			Sensitivity	Specificity	PPV	NPV	Diagnostic accuracy
		Positive	Negative	Total					
IDC (n = 468)	Positive	182	0	182	88.3%	100%	100%	91.6%	94.9%
	Negative	24	262	286					
	Total	206	262	468					
ILC (n = 72)	Positive	12	0	12	85.7%	100%	100%	96.7%	97.2%
	Negative	2	58	60					
	Total	14	58	72					

Table 4 compares the sensitivity, specificity, PPV, NPV, and diagnostic accuracy of intraoperative FS with respect to tumor grade. The sensitivity of FS for grade G1, G2, and G3 tumors was 78.3%, 91.5%, and 90.2%, respectively. Similarly, the diagnostic accuracy of intraoperative FS for grade G1 tumors was 90% while it was 96.7% and 96% for grade G2 and G3 tumors, respectively.

**Table 4:** Comparison of frozen section diagnosis with final (paraffin) section diagnosis for the detection of sentinel lymph node metastasis with respect to tumor grade PPV, positive predictive value; NPV, negative predictive value

Tumor grade	Frozen section diagnosis	Final (paraffin) section diagnosis			Sensitivity	Specificity	PPV	NPV	Diagnostic accuracy
		Positive	Negative	Total					
Grade 1 (n = 100)	Positive	36	0	36	78.3%	100%	100%	84.4%	90%
	Negative	10	54	64					
	Total	46	54	100					
Grade 2 (n = 214)	Positive	75	0	75	91.5%	100%	100%	95%	96.7%
	Negative	7	132	139					
	Total	82	132	214					
Grade 3 (n = 226)	Positive	83	0	83	90.2%	100%	100%	93.7%	96%
	Negative	9	134	143					
	Total	92	134	226					

We compared the false-negative rate of FS with the final (paraffin) section diagnosis with respect to age, tumor grade, histological type, tumor size, and number of SLNs. The false-negative rate for grade G1 tumors was 21.7%, which was higher than G2 and G3 tumors (8.5% and 9.8%, respectively). Similarly, the false-negative rate for cases where the number of SLNs was more than three was only 5.4%, which was lower than cases with a single and two to three SLNs sent on FS (23.1 and 14.7%, respectively). Similarly, the false-negative rate of intraoperative FS for tumors measuring more than 5cm was 0% as compared to 4.5% and 14.5% for tumors measuring less than or equal to 2 cm, and tumors measuring between 2.1 cm and 5 cm, respectively (Table 5).

**Table 5:** Comparison of the false-negative rate of the frozen section with the final (paraffin) section diagnosis with respect to clinicopathological characteristics IDC, infiltrating ductal carcinoma; ILC, infiltrating lobular carcinoma; SLN, sentinel lymph node

Clinicopathological characteristics	Values
	False-negative rate
Age groups	
≤50 years, %	11.4
>50 years, %	12.5
Tumor grade	
Grade 1, %	21.7
Grade 2, %	8.5
Grade 3, %	9.8
Tumor size (cm)	
≤2 cm, %	4.5
2.1-5 cm, %	14.5
>5 cm, %	0
Histological type	
IDC, %	11.7
ILC, %	14.3
Number of SLNs	
1, %	23.1
2-3, %	14.7
>3, %	5.4

## Discussion

In this study, we noted a good overall diagnostic accuracy of FS for detecting breast cancer metastasis to axillary SLNs. The sensitivity of FS for detecting ILC metastasis to axillary SLNs was comparable to IDC (85.7% vs. 88.3% for ILC and IDC, respectively). Alternatively, there was a substantial difference in the false-negative rate of FS for detecting SLN metastasis with respect to tumor grade and the number of SLNs sent on FS intraoperatively.

Mukhtar et al. studied the false-negative rate of SLN biopsy for patients with ILC. They found no significant difference compared with other histological types; however, they concluded that an increasing number of SLN improves the diagnostic accuracy of SLN biopsy for detecting breast cancer metastasis [15]. Similar findings were noted in our study. Horvath et al. compared the diagnostic accuracy of intraoperative FS for 131 consecutive cases of ILC with 133 cases of IDC. They found no significant difference in terms of sensitivity, specificity, PPV, and NPV [16]. Concordant with these findings, in our study, the difference in the false-negative rate of FS for IDC and ILC was unsubstantial.

There is continuous debate and confusion among surgeons regarding the use of intraoperative FS for ILC of the breast. Guo R et al. in a study involving 707 cases of ILC reported a false-negative rate of 8.97%. They emphasized that in their study, 183 of 196 cases with positive SLNs were clinically node-negative. Moreover, cases with false-negative FS were older with a fewer number of SLNs removed than patients with a false-negative diagnosis [17]. These results further emphasize a need to evaluate more SLNs on FS than just a single lymph node.

The American College of Surgeons Oncology Group Z0011 (ACOSOG Z0011) randomized controlled trial evaluated the difference in overall survival of patients with SLN dissection (without axillary dissection) versus complete axillary clearance in patients undergoing breast conservation surgery. The trial enrolled patients with one or two positive SLNs. The median follow-up period was 9.3 years. The trial concluded that in patients with tumor stage T1 and T2 breast cancers undergoing breast conservation surgery, with clinically negative SLNs and histologically less than three positive SLNs should not undergo complete axillary clearance, as there was no significant difference in survival with or without axillary dissection in these patients [18]. In our series of patients, axillary dissection was not performed in patients with one or two positive SLNs on FS in patients undergoing breast conservation surgery. Alternatively, in patients undergoing mastectomy, axillary dissection was performed with even a single positive SLN macrometastasis on FS.

The limitations of our study include a fewer number of ILC cases than IDC and single-institution data. Therefore, we recommend large-scale prospective studies, including other histological subtypes of breast cancer evaluating the impact of other pathological and clinical factors impacting the sensitivity of intraoperative FS for detecting breast cancer metastasis to axillary SLNs.

## Conclusions

Intraoperative FS is a useful technique to evaluate breast cancer metastasis to axillary SLNs. In our study, we noted that the difference in the diagnostic accuracy of FS for detecting IDC metastasis to axillary SLNs was unsubstantial as compared with ILC. However, the false-negative rate for low-grade tumors was higher than for high-grade tumors. Moreover, increasing the number of SLNs sent intraoperatively for FS improves the sensitivity for detecting breast cancer metastasis to axillary SLNs.
